# The Human Neonatal Gut Microbiome: A Brief Review

**DOI:** 10.3389/fped.2015.00017

**Published:** 2015-03-05

**Authors:** Emily C. Gritz, Vineet Bhandari

**Affiliations:** ^1^Division of Perinatal Medicine, Department of Pediatrics, Yale Child Health Research Center, Yale University School of Medicine, New Haven, CT, USA

**Keywords:** newborn, preterm, microbiota, probiotics, dysbiosis

## Abstract

The field of genomics has expanded into subspecialties such as metagenomics over the course of the last decade and a half. The development of massively parallel sequencing capabilities has allowed for increasingly detailed study of the genome of the human microbiome, the microbial super organ that resides symbiotically within the mucosal tissues and integumentary system of the human host. The gut microbiome, and particularly the study of its origins in neonates, has become subtopics of great interest within the field of genomics. This brief review seeks to summarize recent literature regarding the origins and establishment of the neonatal gut microbiome, beginning *in utero*, and how it is affected by neonatal nutritional status (breastfed versus formula fed) and gestational age (term versus preterm). We also explore the role of dysbiosis, a perturbation within the fragile ecosystem of the microbiome, and its role in the origin of select pathologic states, specifically, obesity and necrotizing enterocolitis (NEC) in preterm infants. We discuss the evidence supporting enteral pre- and pro-biotic supplementation of commensal organisms such as *Bifidobacterium* and *Lactobacillus* in the neonatal period, and their role in the prevention and amelioration of NEC in premature infants. Finally, we review directions to consider for further research to promote human health within this field.

## Introduction

The evolution of the field of genomics, spurred largely by the Human Genome Project, has given rise to metagenomics, or “the application of modern genomics techniques to the study of communities of microbial organisms directly in their natural environments” (1). This burgeoning area of study has resulted in significant advances in whole-genome analysis techniques that have greatly facilitated the study of the human microbiome.

The human microbiome is a microbial community best described as “the sum of all microbial life living in or on the human body” (2). It is an entity that has wide-reaching metabolic, nutritional, and immunological effects on the host, and as such has generated a great deal of interest within the biomedical research community. The microbiome evolves within a healthy host from birth to death, constantly fine-tuning it to maintain a homeostatic balance with the host’s immune system. Continued evolution of the human microbiome after birth is governed by host factors such as both the adaptive and innate immune system, as well as external factors such as diet, medication and toxin exposure, and illness (3, 4). Of particular interest is the study of the gut microbiome, its evolution beginning *in utero* and across the lifespan, its effect on promotion of health, and its role in the development of disease.

The purpose of this brief review is to introduce the reader to the concept of the human microbiome and to summarize recent literature specifically regarding the neonatal gut microbiome, from its establishment at birth through its evolution during early infancy. We review differences in microbial colonization and immune function of the intestinal tract in healthy full-term newborns compared with their preterm very low birth weight (VLBW) (birth weight <1500 g) counterparts and the implications for development of disease when the microbiome is disrupted (dysbiosis). Finally, we describe the role of probiotic use as a potential factor involved in governing development of a “healthy” gut microbiota and discuss areas of further research warranted to deepen our understanding of this complex ecosystem and its role in human health and disease.

## Establishment and Evolution of the Neonatal Gut Microbiome

**Figure 1:**
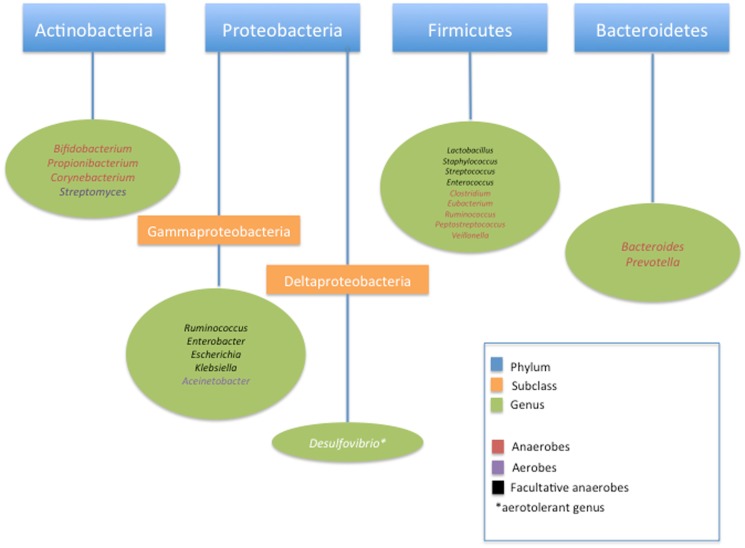
**Classification of common bacteria found in neonatal intestinal microbiome**.

The first, and most important, contribution to the genesis of the microbiome is vertical transmission of maternal microbiota. Colonization of mucosa in the digestive, respiratory, urogenital tracts, as well as the skin begins at, or perhaps even before, the time of birth when a newborn is exposed to a mother’s microbiota. It was previously thought that the *in utero* environment was largely sterile and that a fetus was not colonized with bacteria until the time of birth. Recent studies suggest the presence of a microbiome within the placenta as well as fetal meconium, suggesting that the colonization process begins well before delivery. Aagaard et al. have recently characterized a placental microbiome profile, composed of non-pathogenic commensal microbiota from the Firmicutes, Tenericutes, Proteobacteria, Bacteroidetes, and Fusobacteria phyla which, interestingly, shares some similarities with the human oral microbiome (5). They observed that in the first week of life the full-term neonatal gut microbiome is largely colonized by members of the Actinobacteria, Proteobacteria, Bacteroidetes, and, much less, Firmicutes phyla (Figure 1) (5, 6). This is contrasted with the previously described finding that neonates weighing <1200 g have a gut microbiome dominated by members of both Firmicutes and Tenericutes phyla (5, 7, 8). This evidence of early colonization of the neonatal gut microbiome so close to the time of birth suggests that there may be exposure to an antenatal source of commensal bacteria, such as the placenta, and this seeding may vary by length of gestation (5). It is well known that as fetuses become more neurologically mature, they begin to swallow large amounts of amniotic fluid, particularly during the third trimester of pregnancy. If the uterine environment is colonized with its own microbiota, as is suggested in recent studies, then the fetal gut may in turn become colonized by these organisms. Recent studies suggesting that meconium is not sterile support this theory (9). Ardisonne et al. evaluated meconium samples from neonates and found that bacterial species in meconium were shared with organisms found in the amniotic fluid (10).

As such, the gastrointestinal (GI) tract has the greatest diversity and abundance of microbes and evidence is mounting that it becomes colonized antenatally. Approximately 100 trillion organisms – which is 10 times the total number of cells in the human body – typically comprise the gut microbiota and the majority of these populate the distal ileum and colon. More than 99% of the gut microbiota is anaerobes (6, 11). Interestingly, each individual’s microbiome is populated by only 15% of the 1000 plus species of intestinal bacteria already described, leading to significant inter-individual variability of the microbiome (4, 12). Eckburg et al. examined 13,355 prokaryotic ribosomal RNA gene sequences from intestinal mucosal tissue samples and fecal samples of healthy adult subjects and observed that of 395 bacterial phylotypes identified, 244 were novel, and 80% represented sequences from species that have not been cultivated (13).

It is easy to conceptualize then that the collective genome of the intestinal microbiota is >100 times the number of genes in the human genome; as such, there are a significant potential number of commensal antigens for the host immune system to encounter and to respond. This exceeds the number of self and pathogen-based antigens a human will encounter in their lifetime (12).

Infants born via vaginal delivery have intestinal colonization reflective of maternal vaginal flora such as *Lactobacillus* and *Prevotella* species. Infants born via Cesarean delivery are colonized by epidermal rather than vaginal species, such as *Clostridium*, *Staphylococcus*, *Propionobacterium*, and *Corynebacterium* and they have a deficiency of anaerobes with lower numbers of *Bacteroides* and *Bifidobacterium* when compared to vaginally born infants (14–18). Thus, the mode of delivery appears to have an influence on the diversity and function of an infant’s microbiota, which can persists for months and, perhaps longer, after birth. Jakobsson et al. demonstrated that full-term infants delivered via Cesarean section lacked or displayed delayed gut colonization by members of the Bacteroidetes phylum by up to 1 year, with an overall lower total microbial diversity (19). Other studies have also shown persistent differences in intestinal microbial colonization between Cesarean-delivered and vaginally delivered children as far as 7 years of age (20).

Further development of the neonatal gut microbiome after birth, regardless of mode of delivery is governed by interaction between the microbiota and the host’s immune system. The progression of how this evolves remains incompletely characterized and many questions remain surrounding the true origins of the microbes that colonize the neonatal gut and what factors underlie inter-infant differences in gut microbiota, Palmer et al. evaluated stool samples of 14 healthy full-term infants using rDNA microarray technology to define their gut microbial profiles over the course of their first year of life and found that while at a phylum level diversity of the analyzed samples was mostly limited to *Flexibacter*–*Cytophaga*–*Bacteroides*, Proteobacteria, and Firmicutes and Actinobacteria; there was a remarkable level of inter-individual variation from baby to baby over the course of the study (21). They noted an overall earlier appearance of aerobes such as *Staphylococcus*, *Streptococcus*, and *Enterobacteria*; a later appearance of anaerobes such as *Eubacteria* and *Clostridium*, and variable timing of the emergence of *Bacteroides*, which ultimately established a presence in all babies by age 1 year. Transient colonization was noted at varying time points by other organisms including *Prevotella*, *Acinetobacter*, *Desulfovibrio*, *Veillonella*, and *Clostridium perfringens* (21). In contrast, a 2012 study by Turroni et al. demonstrated *Bifidobacteria* species as a predominant component of the term infant gut microbiota, challenging the notion that the infant gut contains low numbers of *Bifidobacterium* (22). The differences seen between these studies highlights the need for cautious further investigation to best characterize the infant gut microbiome. Nevertheless, gut colonization patterns established within the first week of life are thought to have effects on the composition of the individual’s future gut microbiota via a variety of factors (17, 19, 23, 24). The term infant’s gut microbiome undergoes rapid maturation over the first year of age and is securely established in an adult form by 3 years of age. An individual’s ultimate adult gut microbiome profile is likely governed by an elucidated interplay between initial colonizing microbiota, genes, normal gut development, diet, and environment (14, 21, 25, 26).

Once established, the intestinal microbiome engages in a symbiotic relationship with its host. The neonatal immune system will rapidly mature secondary to influence of microbiota, diet, exposure to new microbes, xenobiotics, and other environmental exposures (12). The organisms that comprise the microbiota benefit from the warm, nutrient-rich environment afforded by the gut. This allows for optimum growth within a stable ecosystem. The human, in turn, benefits from activities of the microbiota that primarily allow for an increased digestive capacity and an ability to harvest nutrients from food. Weaning from breast milk or formula and introducing solid foods causes a shift in composition of the neonatal gut microbiome and leads to increased counts of *Bacteroides*, *Clostridium*, and anaerobic species of *Streptococcus* but decreased numbers of *Bifidobacterium*. This shift in bacterial makeup leads to expression of bacterial genes that are involved both in degradation of xenobiotic compounds, vitamin biosynthesis, and production of other metabolites such as butyrate and acetate (21, 25, 27, 28). In addition to these pro-nutrient effects, the intestinal microbiome can limit nutrient resources available to pathogens, specifically by out-competing them for metabolic resources and for physical space (12, 27). Gut microbiota also aid in development of barrier function, integrity, and systemic immune function. This includes formation of a tolerant state between gut organisms and the immune system and is thought to affect tight junction structure and function (3, 29). Generation of pro-inflammatory or anti-inflammatory responses as a result of exposure to bacteria include activation or deactivation of toll like receptors, T-lymphocyte activation, and triggering secretion of pro- and anti-inflammatory interleukins and cytokines, as well as activation of B-cells within the mesenteric lymph node system (3, 25) – the scope of which is beyond this present review. Overall, these arms of the innate and adaptive immune system interact with the microbiota to establish normal digestive capabilities, gut motility, immune tolerance to foods and certain microbial antigens, and protection against pathogens (25). The exact role of the neonatal gut microbiome in the development and maturation of the infant immune system, as well as the influence of the neonatal immune system on governance of the fledgling gut microbiome is still poorly understood from a mechanistic standpoint and continues to be a growing area for further research (27).

## The Role of Nutrition in Development of the Full-Term Neonatal Gut Microbiome

Nutrition, be it breast milk or formula, has been demonstrated to play a major role in early colonization patterns of the neonatal gut microbiota. Healthy, full-term breastfed infants receive a mix of nutrients, bacteria, and antimicrobial proteins, such as carbohydrates, fatty acids, and lactoferrin along with secretory IgA (sIgA) from their mother’s milk that will affect the milieu within which their own microbiota will develop (27). Oligosaccharides, glycoconjugates, and natural components of human milk are also thought to prevent the attack of enteropathogens and stimulate growth of *Bifidobacterium* (4, 30, 31). These human milk oligosaccharides are known to directly interact with the surface of pathogenic bacteria, and various oligosaccharides in milk are believed to inhibit the binding of pathogens and toxins to host cell receptors (32). Other constituents of human milk, such as interleukin-10, epidermal growth factor, transforming growth factor-β1, and erythropoietin, can represent important mediators in the inflammatory response against bacterial pathogens within the gut (4).

Live bacteria are also found in human milk, including *Staphylococcus*, *Streptococcus*, *Bifidobacterium*, and *Lactobacillus* (33). Several associated factors influence the dynamic composition of the breast milk microbiota, including maternal health and mode of delivery (34). At least some of the bacteria present in the maternal gut is thought to reach the mammary gland through an endogenous route, the so-called enteromammary pathway and likely contributes to the bacterial composition of breast milk (17). The composition of breast milk bacteria becomes increasingly less diverse, beginning with typical skin- and enteric-type organisms in colostrum to less diverse flora with greater infant oral and skin microbiota as lactation progresses (35). Non-digestible carbohydrates found in breast milk ferment in the colon and promote further growth of probiotic *Bifidobacterium* and *Bacteroides* species (27). Interestingly, it has also been noted that during lactation, the cells of the maternal intestinal lymphoid tissue travel via the lymphatic and vascular circulations to the breast, facilitating the transfer of maternal intestinal and mammary skin microbiota to the breast-fed newborn (4, 36).

Formula-fed infants are exposed to a different array of carbohydrates, bacteria, and nutrients, which leads to different colonization patterns and immunomodulatory effects on their developing gut microbiota. In contrast to human milk oligosaccharides as mentioned earlier in this section, oligosaccharides currently added to infant formula are structurally different from those naturally found in human milk and, therefore, are unlikely to mimic some of the structure-specific effects on the gut that are seen in breast-fed neonates (32). It has been demonstrated that term breastfed infants have a gut microbiota dominated by species of *Bifidobacterium* but decreased *Enterobacteria*. Formula-fed counterparts, regardless of milk-based or soy-based formula composition, have a gut microbiota comprising a more diverse array of bacteria including *Escherichia coli*, *Clostridium difficile*, *Bacteroides*, *Prevotella*, and *Lactobacillus* (14–16, 27, 37–40) (Table 1). Interestingly, even relatively small amounts of formula supplementation of breast-fed infants will result in shifts from a breast-fed to a formula-fed pattern (41, 42). A 2011 study by Hascoet et al. demonstrated, however, that infants, who received a formula low in phosphate and protein, comprise mainly whey protein in an attempt to provide a composition closer to human milk, developed a stool microbiota profile similar in *Bifidobacteria* composition to that for breast-fed infants (43). This suggests that even in infants not receiving exclusive breast milk, a breast-fed gut microbiota could be achieved, in part at least, by supplementing with a type of formula having a composition similar to breast milk.

**Table 1:** **Major differences in neonatal gut colonization by type of feeding**.

Breast fed	Formula fed
*Bifidobacteria*^a^	*Bifidobacteria* species
*Enterobacteria* species	*Escherichia coli*
	*Clostridium difficile*
	*Bacteroides* species
	*Prevotella* species
	*Lactobacillus* species

*^a^Breast-fed infants have more colonization with *Bifidobacteria* species than their formula-fed counterparts*.

In breast-fed infants, transmission of sIgA from the mother is reflective of her own microbiota and confers a protective effect against pathogens that could lead to dysbiosis, the disruption of a healthy, functional infant microbiome. sIgA is also thought to shield the neonatal immune system from its own microbiota while host defenses are maturing. The purported mechanisms for this are binding of microbial antigens by sIgA and activation of the host’s innate immune system in a more “tolerogenic” mode upon antigen exposure. This promotes formation of regulatory immune networks that further govern development and function of the gut microbiome (12).

This difference in colonization and transmission of immune-modulating factors between breast and formula-fed infants may have far reaching effects over the course of a human’s life as it may impact disease risk. The gut microbiota’s metabolic activity, specifically its ability to extract nutrients from food consumed by its host, may have a variable effect, depending on species diversity and composition, on an infant’s ability to store and utilize energy efficiently (14). An increasingly investigated avenue is the relationship between alterations in the gut microbiome and its possible involvement in the development of disease later in life. A prime example of this is the obesity epidemic, which may begin as early as the perinatal period. While this epidemic is thought to be primarily due to excessive consumption of carbohydrates and fats coupled with decreased physical activity, childhood obesity has been, “in part, attributed to the fetus exposure to unfavorable conditions (e.g., nutritional and hormonal dysfunctions) in the uterine life, which can then exert a strong impact on the subsequent development, structure, and function of the child organism” (4, 44). This phenomenon, which can extend to perinatal and postnatal age, is known as disease programing during the development phase (4, 45).

As previously noted, the gut microbiota serves a critical metabolic function for its host. In particular, it enables digestion of otherwise indigestible carbohydrates and triggers activation of lipoprotein lipase. This leads to glucose absorption and storage of fatty acids and thus to excessive weight gain. Increased numbers of Firmicutes and decreased Bacteroidetes in the gut microbiota have also been shown in experimental animal models to predispose toward excess energy storage and obesity (46, 47). Production of metabolites such as short chain fatty acids such as butyrate and acetate by early commensal gut microbiota may play a role in epigenetic alteration of gut epithelium and immune function that predispose to diseases like obesity (25, 48). Kalliomaki et al. demonstrated increased *Bifidobacterium* in fecal samples during the first year of life in children who remained normal weight at 7 years of age when compared to children who became overweight (46). Luoto et al. later reinforced this finding by demonstrating in a 2011 longitudinal study of 30 obese children that obese subjects had lower levels of *Bifidobacterium* as infants when compared to normal weight counterparts (49). VLBW infants are paradoxically at increased risk for development of obesity later in life possibly due to metabolic programing that predisposes toward energy storage even when nutrients are not in short supply. An alternative hypothesis, which could explain this predisposition toward obesity in this population centers around altered nutrient processing and utilization and immune regulation, has a function of altered gut microbiota (25). To date, little research has been done to evaluate the effect of composition of the neonatal gut microbiota, term or preterm, its relationship to early nutritional status, and its effect on later development of obesity and other pro-inflammatory disease states. Given obesity’s long-reaching effects across the lifespan, seeking to further understand its origins and metabolic underpinnings, perhaps beginning as early as the neonatal period, remains an important focus of continued investigation.

## The Preterm Gut Microbiome

Preterm infants, particularly VLBW infants, are at a disadvantage when it comes to development of a healthy microbiome. Factors contributing to this are not limited to their gut immaturity, and also include preterm rupture of membranes, maternal infection, increased incidence of Cesarean delivery, perinatal and postnatal broad-spectrum antibiotic exposure as well as exposure to other gut-modifying medications such as H2 blockers, altered gut motility, periods of fasting, intensive care infection control standards and selection for resistant microbes, and decreased exposure to human milk (17, 25, 29, 50). Given these factors, it seems likely, and has indeed been shown to be true that a preterm infant’s gut microbiota has reduced microbial diversity coupled with an increase in colonization with pathogenic organisms (17, 51). Additionally, the preterm gut microbiome is less stable compared to that of term counterparts and is also thought to be delayed in transition to an adult colonization pattern (3, 14, 25, 29). Arboleya et al. demonstrated this by comparing full-term breastfed vaginally delivered infants with preterm infants with regard to differences in representation of 18 microbial groups within gut flora. They demonstrated that when compared with full-term infants, preterm infants showed increased populations of facultative anaerobes such as *Enterococcus*, *Enterobacter*, and *Lactobacillus*, increased numbers of *Staphylococcus*, and decreased numbers of anaerobes like *Bifidobacterium*, *Bacteroides*, and *Atopobium* (3, 29, 52). A 2007 study by Butel et al. found that healthy full-term breastfed infants are colonized by *Bifidobacterium* by day 7 of life, whereas preterm infants are not. Interestingly, they also suggest that there may be gestational age thresholds for colonization with certain microbes – 33 weeks appears to be the milestone for appearance of *Bifidobacterium* species, the organism most commonly implicated in development and maintenance of a healthy gut microbiome (53). Very recently, LaRosa et al. used 16s rRNA gene pyrosequencing to show a reproducible longitudinal succession of bacterial classes from Bacilli to Gammaproteobacteria to Clostridia in 58 VLBW infants born at 23–33 weeks gestational age in a single neonatal intensive care unit (NICU). They found that this evolution was marked by periods of “abrupt population changes” that ultimately achieve a common endpoint wherein by 33–36 weeks postconceptional age, study infants consistently had gut microbiota predominantly colonized by anaerobes, particularly Clostridia, which approached levels of older individuals. Of note, mode of delivery, antibiotic exposure, mode of feeding, and age of infants at time of sampling only affected the rate of progression toward an anaerobic-dominated microbiota in the study infants, and not the sequence to achieve it (54). A 2013 study of longitudinal development of the preterm gut microbiome in twins also demonstrates that they share similar gut microbiome development even within the complex, multiexposure environment of a NICU suggesting that in preterm infants, development of the gut microbiome may also be influenced by genetics (51). While the evolution of the term infant gut microbiome has been somewhat characterized, to date, there are still few prospective studies of the evolution of the preterm, VLBW gut microbiome to assess if there is a characteristic patterned succession and time course of microbial colonization for this group from birth through early childhood.

In addition to altered microbial diversity as described above, premature infants are both qualitatively and quantitatively immunodeficient, owing to their underdeveloped immune systems. They have suboptimal gut epithelial cell barrier function at baseline, predisposing them to invasion by pathogens that in turn can trigger exaggerated inflammatory responses by their still-developing immune system that may lead to disease processes such as necrotizing enterocolitis (NEC) (16, 55). This immune dysfunction, coupled with low diversity of gut microbiota and possibly an overall predominance of pathogenic bacteria within the preterm intestinal microbiome has been noted in at-risk preterm neonates with life threatening *Enterobacter* and coagulase negative *Staphylococcus* sepsis, and is a prime example of dysbiosis (14, 56, 57).

## Dysbiosis

Even after the microbiome is well established in healthy infants, dysbiosis, or shifts in microbial composition or diversity, can occur in the setting of dietary changes, antibiotic exposure, or infection. Dysbiotic conditions can favor invasion and growth of pathogenic species and can disrupt the finely tuned regulatory circuits of the immune system that maintain a system of pro- and anti-inflammatory checks and balances. The neonatal microbiome, in healthy full-term infants and especially in preterm infants given its dynamic nature, is fragile and impressionable. As such, the microbiome is extremely susceptible to external influences that can dramatically affect the short- and long-term health of the host. The development of NEC in the preterm population is a multifactorial, devastating, and as yet poorly understood disease process. A link between NEC and a microbial etiology has been recognized for decades and has been corroborated by outbreaks in NICUs, the presence of pneumatosis intestinalis as a likely byproduct of bacterial fermentation, and the often concomitant presence of bacteremia (58). As such, NEC is increasingly thought to be, at least in part, related to a perturbation of intestinal immune homeostasis, and a generalized disturbance of normal colonization patterns within the developing gut, rather than growth of a single pathogen (59, 60). The advent of a variety of techniques for metagenomic analysis of the developing human gut microbiome has given way to studies investigating whether there is a signature microbial pattern that predisposes to or heralds the onset of NEC. As summarized by Berrington et al., recent analyses of microbiomic data from preterm infants with NEC show great variability in proposed dysbiotic growth patterns (3). Some studies implicate increased Proteobacteria and decreased Firmicutes in the development of NEC (61), while others note more than one pattern of dysbiosis within a given neonatal cohort. Morrow et al. noted both Firmicute dominant and Proteobacteria dominant patterns within their cohort of preterm infants, the former being associated with earlier presentation of NEC, the latter with later presentation (62). Alternatively, other groups have found no differences in microbiota between NEC-affected patients and healthy controls (63).

## The Role of Pre- and Pro-Biotics in Development of the Neonatal Gut Microbiome

Given the relative instability and impressionability of the developing gut microbiome in early life, coupled with its purported disease prediction, detection, and treatment benefits, it seems logical to explore avenues within which evolution and maintenance of a healthy gut milieu can be promoted. One such intervention that has gained overwhelming popularity over the last two decades, but that remains a controversial topic, is the use of pre- and pro-biotics. Given that an infant’s health and well-being are tightly linked to the development of the intestine and its digestive and immune capacities, it would seem logical that manipulation of the microbiota with the use of pre- and/or pro-biotic nutritional supplementation at an early stage could have a high and long-lasting impact (11).

Prebiotics are “non-digestible food ingredients that selectively stimulate the growth or activity of anaerobic/microaerophilic flora (*Bifidobacterium*/*Lactobacillus*) in the colon of mammals” (55). Some studies also suggest that in addition to promoting growth of commensal organisms like *Bifidobacterium* and *Lactobacillus*, prebiotics may also improve intestinal motility and gastric emptying (64–66). These sugars are present in several food sources, including breast milk and commercially available infant formulas. Lactoferrin is a natural component of human milk with antimicrobial, immunostimulatory, and immunomodulatory properties and has been shown to promote a gut environment in neonates that predisposes toward colonization with favorable bacterial such as *Bifidobacterium* and *Lactobacillus* species (67). Mastromarino et al. recently demonstrated that early high levels of fecal lactoferrin in neonates may contribute to the immunologic maturation and overall health of the newborn by promoting colonization with *Bifidobacterium* and *Lactobacillus* species, particularly in preterm infants (67). Inulin, lactulose, and short chain fructo-oligosaccharides (FOS) and galacto-oligosaccharides (GOS) are several other well-studied prebiotics in humans, but their use, efficacy, and safety in the neonatal population, particularly the preterm neonatal population, is not well studied (55).

Probiotics are live microorganisms that when administered in adequate amounts, ideally confer a health benefit on the host – as defined by the World Health Organization (WHO). Probiotic supplementation in the preterm neonatal population is purported to promote acquisition of normal commensal gut flora in these compromised hosts and to confer a protective effect against dysbiotic conditions such as NEC.

Several recent clinical trials and meta-analyses on this subject suggest that probiotic administration for NEC prevention is overall thought to be safe and effective (68). It is important to note, however, that the significant degree of heterogeneity among studies included in these meta-analyses renders conclusions about the safety and efficacy of probiotics in the vulnerable preterm population up for debate (55, 69). To date, virtually all trials use combinations of different probiotics. Common probiotic preparations given to neonates include *Lactobacillus* and *Bifidobacterium*. Probiotic preparations that recently have been studied in the neonatal population are summarized in Table 2. Of note, of the 12 trials described in the table, 4 of them cite no difference in NEC prevention between treatment and placebo groups. Further examination of Table 2 highlights a notable degree of study to study variability in terms of inclusion criteria, type, and dose of probiotic administered. In addition, in one study, a fungal species (*Saccharomyces*) is used as a probiotic. This diversity in design and execution of clinical studies of probiotics in neonates is also evident in the recent meta-analyses by Deshpande et al., and Wang et al., a systematic review by Mihatsch et al. and a 2014 Cochrane review (69–72). These factors may, in part, underlie the variability of outcomes related to NEC prevention. Janvier et al. has commented that across recent trials “subgroup analyses demonstrate little difference in the effects of probiotics between those containing lactobacilli, those containing just *Bifidobacteria*, and those containing a mixture, although there are trends suggesting that a mixture of different organisms may be more effective than a single species,” but this continues to require further study (73, 74).

**Table 2:** **Summary of species used in recent probiotic therapy trials in preterm infants**.

Reference	Year	Study type	Study size	Inclusion criteria	Probiotic species studied^a^	Daily dose	Outcomes
Janvier et al. (73)	2014	Cohort	294	<32 weeks Gestational age	*Bifidobacterium breve*	2 × 10^9^ CFU	Significantly decreased incidence of NEC in subjects receiving probiotics
					*Bifidobacterium bifidum*		No effect on in incidence of death
					*Bifidobacterium infantis*		No effect on rates of healthcare associated infection
					*Bifidobacterium longum*	
					*Lactobacillus rhamnosus*	

Oncel et al. (75)	2014	RCT	424	<32 weeks Gestational age	*Lactobacillus reuteri*	1 × 10^8^ CFU	No effect of probiotic therapy on overall rates of NEC and/or death
							Noted decreased feeding intolerance in infants receiving probiotic therapy

Jacobs et al. (76)	2013	RCT	1099	<32 weeks Gestational age	*Bifidobacterium infantis*	1 × 10^9^ CFU each	Significant reduction in rates of NEC (Bell stage ≥2)
					*Bifidobacterium lactis*		No effect on rates of late-onset sepsis
					*Streptococcus thermophilus*		No effect on overall neonatal mortality

Serce et al. (77)	2013	RCT	208	<32 weeks Gestational age	*Saccharomyces boulardii*	1 × 10^9^ CFU	No effect on incidence of NEC or sepsis
				Birth weight <1500 g	

Fernandez-Carrocera et al. (78)	2013	RCT	150	Preterm infants	*Bifidobacterium infantis*	2.76 × 10^7^ CFU	No differences were detected in terms of NEC risk reduction
					*Lactobacillus rhamnosus*	4.4 × 10^8^ CFU	Decreased frequency of NEC
					*Lactobacillus acidophilus*	1 × 10^9^ CFU	Significantly decreased combined risk of NEC or death in infants receiving probiotic therapy
					*Lactobacillus casei*	1 × 10^9^ CFU	
					*Lactobacillus plantarum*	1.76 × 10^8^ CFU	
					*Streptococcus thermophilus*	6.6 × 10^5^ CFU	

Rojas et al. (79)	2012	RCT	770	Birth weight <2000 g	*Lactobacillus reuteri*	1 × 10^8^ CFU	40% Overall decrease in NEC cases, but not significant
							Decreased feeding intolerance in infants receiving probiotic therapy

Al-Hosni et al. (80)	2012	RCT	101	Birth weight <1000 g	*Lactobacillus rhamnosus*	5 × 10^8^ CFU each	No effect on incidence of and mortality due to NEC
					*Bifidobacterium infantis*		Probiotic supplemented feedings improved growth velocity

Braga et al. (81)	2011	RCT	231	Birth weight <1500 g	*Bifidobacterium breve*	3.5–3.7 × 10^7^ CFU	Significant decrease in incidence of NEC (Bell stage ≥2)

Samanta et al. (82)	2009	RCT	186	<32 weeks Gestational age	*Bifidobacterium bifidum*	2.5 × 10^9^ CFU each	Significant decrease in incidence of and death due to NEC
				Birth weight <1500 g	*Bifidobacterium infantis*		Decreased feeding intolerance in infants receiving probiotic therapy
					*Bifidobacterium longum*	
					*Lactobacillus acidophilus*	

Lin et al. (83)	2008	RCT	234	<34 weeks Gestational age	*Bifidobacterium bifidum*	1 × 10^9^ CFU each	Significant decrease in incidence of NEC or death
				Birth weight <1500 g	*Lactobacillus acidophilus*	

Lin et al. (84)	2005	RCT	367	Birth weight <1500 g	*Bifidobacterium infantum*	1 × 10^9^ CFU each	Significant decrease in incidence of NEC or death
					*Lactobacillus acidophilus*	

Bin Nun et al. (85)	2005	RCT	145	Birth weight <1500 g	*Bifidobacterium bifidus*	1 × 10^9^ CFU each	Significant decrease in incidence and severity of NEC
					*Bifidobacterium infantum*	
					*Streptococcus thermophilus*	

*^a^All species used as combination preparations of probiotics*.

*RCT, randomized clinical trial*.

Another important consideration in evaluating whether or not the use of probiotics in this population is warranted is the effective dose and duration of treatment. In order to produce health effects, probiotic organisms need to be able to survive within the GI tract and persist at high levels within the intestine (86), but the minimum effective dose at which this can be achieved remains to be determined. Historically, the ability to reliably determine what organisms colonize the intestinal tract has been limited by use of plate culture methods that only allow identification of a limited number of organisms within the vast microbial population of the gut (87). The advent of highly sensitive techniques such as quantitative PCR (qPCR) enables detection and quantification of as little as a single copy of target DNA. This, coupled with improved computer-based comparative metagenomic techniques, allows for better characterization of the organisms that colonize and comprise the intestinal microbiome (88).

A 2003 study by DeChamps et al. evaluated the colonization ability of *Lactobacillus rhamnosus* Lcr 35 at doses of 10^8^, 10^10^, 10^12^ CFU/day over a 3-week period in 12 healthy adults and found no relation between the average number of cultured *Lactobacillus* Lcr 35 CFU in feces and doses ingested by study subjects (86). They demonstrated that following a 3-week post-treatment period wherein no probiotics were administered to subjects, the CFU levels of *Lactobacillus* in fecal samples were slightly decreased, albeit similar to levels observed immediately following probiotic treatment (86). A culture-based 2008 study by Panigrahi et al. evaluated healthy newborns >35 weeks gestation who were given a synbiotic (*Lactobacillus plantarum* and fructooligosaccharide) for 7 days and found that the synbiotic produced rapid colonization of the infant GI tract (within 3 days of administration) and infants remained colonized, at decreasing rates, for several months after therapy was stopped (89). Costa et al. evaluated 61 healthy adults who received daily *L. plantarum* for differing periods of time. *L. plantarum* levels were monitored over time using qPCR. They noted a discrepancy between the daily intake of *Lactobacillus* (2 × 10^11^ cells/dose) and levels detected in feces (10^4^ cells/g), but noted that they were unable to account for the distribution or fate of bacteria within the GI tract following ingestion. Interestingly, they noted that following cessation of probiotic therapy, detection decreased to pre-intervention levels, suggesting that there is the possibility that certain probiotics may not persist permanently (87). Tobin et al. detected via qPCR the initial presence of 3/3 probiotic species in 83% of premature infants who ingested probiotics after a week of treatment, but noted colonization by only one species in 75% of patients four weeks after cessation of probiotic therapy (90). Other studies on probiotic gut colonization in both preterm and full-term neonates have demonstrated lower levels of colonization (91–93) as well as transient colonization (94). These results suggest that the effectiveness of different probiotic preparations in colonizing the gut may underlie variability in clinical trial results as noted in Table 2 (89). To date, no clinical trials have correlated probiotic colonization with efficacy in the preterm neonatal population, a population thought by many to be more amenable to colonization (87) but with the advent of higher resolution techniques such as qPCR to help characterize the composition of the gut microbiome, this should be investigated further.

Proposed mechanisms of probiotic action at the level of the gut epithelium include enhanced epithelial barrier function, enhanced mucosal IgA responses, direct antagonism against pathogens, competitive exclusion of pathogens, prevention of apoptosis, production of anti-inflammatory cytokines, and down-regulation of pro-inflammatory pathways such as activation of nuclear factor κB (55, 64, 68, 95). The precise mode of action of probiotics is likely strain-dependent, and is difficult to assess due to the overall complexity of the microbiota and its interaction with the immune system (96). *In vitro* and *in vivo* studies have demonstrated that *Lactobacillus* and *Bifidobacterium* exert direct effects on intestinal epithelial barrier function by decreasing intestinal permeability and improving intestinal epithelial resistance (96–99). *In vitro* studies by Karczewski et al. with *L. plantarum* demonstrated a possible role for toll like receptor 2 (TLR2) as one important mediator of epithelial cell barrier integrity (100). This study also suggested that interplay between probiotic species and the gut epithelium may have an immunomodulatory effect by regulating enterocyte cytokine production (96, 100). Martin also suggests that consumption of probiotic species may alter the balance of Th1/Th2 equilibrium within the gut and may lead to prevention or treatment of allergies or infectious diseases (96). Smelt et al. demonstrated that short-term administration of two different lactobacillus strains (*L. plantarum* WCFS1 and *L. salivarius* UCC118) to healthy adult mice induces marked changes in cellular adaptive immune responses in a strain-specific manner (101). *L. plantarum* was shown to increase the frequency of regulatory T cells while decreasing responsiveness of Th2 cells and increasing responsiveness of CD8+ T cells in the spleen and/or mesenteric lymph nodes, suggesting a role for this species in modulating Th2-mediated allergic disease and host response to viral infections that require activation of CD8+ T cells. *L. salivarius* also decreased responsiveness of Th2 cells but had no effect on frequency of regulatory T cells and demonstrated a more modest increase in CD8+ T cell responsiveness (101). The study also demonstrated immunomodulation by *Lactococcus lactis*, a non-probiotic species (101). This strain-specific immunomodulation may, in part, underlie the variability in reported effects of different probiotic species across clinical trials in humans.

Another aspect of the probiotic debate that is of paramount importance is the question of its safety as a therapy for mitigating or preventing disease. An extensive literature review of studies addressing the safety of probiotics by Hempel et al. in 2011 concluded that “there is a lack of assessment and systematic reporting of adverse events in probiotic intervention studies, and interventions are poorly documented” (102). The authors cited several case studies in both adults and pediatric populations describing fungemia (*n* = 33) and bacteremia (*n* = 8) potentially associated with probiotic administration and noted that controlled trials did not routinely monitor for infection of test subjects receiving probiotic therapy (102). Zbinden et al. recently published a case series of three VLBW neonates who developed *Bifidobacterium longum* bacteremia after receiving Infloran, a commonly used commercially available probiotic preparation containing *Lactobacillus acidophilus* and *B. longum* (103). Two of the three cases resulted in a transient bacteremia, the third presented as septicemia in the setting of NEC. In all three cases, the *Bifidobacteria* isolated from patient blood cultures and in the Infloran capsule were genetically and biochemically identical (103). Kitajima et al. reported an interesting incidental finding of cross-contamination, resulting in asymptomatic nosocomial acquisition of probiotic strains by untreated infants in a NICU. Their group reported colonization rates of 73 and 91% in their probiotic group at 2 and 6 weeks’ of treatment, and colonization rates of 12 and 44% in control group infants at the same time points (91). However, it is important to mention that the analysis by Hempel et al. of probiotic administration did not show a statistically significant increased risk of adverse events such as GI illness or infection (102).

There is a strong body of evidence that provides promising evidence in favor of use of probiotics in the preterm population. A 2014 Cochrane review regarding efficacy and safety of probiotics for prevention and amelioration of NEC, acknowledges the aforementioned discrepancies between trials, citing need for improved standardized, head to head studies of type, duration, and amount of probiotic to be used, but still strongly advocates for their use (72). The authors of the review evaluated 24 eligible trials, each with notable variability regarding enrollment criteria, dose, formulation, and feeding regimens between studies. Despite the variability noted above, a meta-analysis of the trial data still demonstrated a significant reduction in the incidence and mortality of severe NEC, despite the degree of variability across studies analyzed (72). Additionally, the included trials reported no adverse events such as systemic infection with organisms found in probiotic preparations used (72). As such, the authors of the review call confidently for a change in practice based on their results. Similar results were previously described in 2010 meta-analysis by Deshpande et al. who evaluated 11 RCTs involving 2176 neonates. They reported that 6.56% of 1082 infants who did not receive probiotics developed NEC compared with 2.37% of 1094 infants who did receive probiotic supplementation and that there was a statistically significant lower relative risk {RR: 0.35 [95% confidence interval (CI): 0.23–0.55] (*P* < 0.00001)} of NEC in the probiotic group. They quoted a number needed to treat (NNT) with probiotics to prevent one case of NEC of 25 (71). No significant heterogeneity across studies was observed in their analysis of the data; however, it is important to remember that adjusting for heterogeneity in such meta-analyses may not be sufficient if different probiotic strains are being used. The analysis also confirmed lack of probiotic effect on incidence of late-onset sepsis in preterm neonates. In light of these results, the authors claimed that it could be considered unethical to deny probiotics from at-risk neonates and advocated for their use without any further placebo-controlled trials (71). A 2012 meta-analysis of 20 RCTs by Wang et al. echoed these results, finding that probiotic administration was associated with a significantly decreased risk of NEC and death from NEC in the VLBW preterm neonatal population, with no difference between treatment and placebo groups when evaluating for risk of sepsis (70). More recently, Janvier et al. published results of a randomized trial describing the effect of probiotic administration on incidence and severity of NEC in a single NICU since July 2011. They administered a probiotic preparation composed of four species of *Bifidobacterium* to 294 infants at <32 weeks’ gestational age, and compared them to 317 infants in a control group. They observed a statistically significant reduction in incidence and severity of NEC, but no effect on incidence of nosocomial infection, once again suggesting a protective role of probiotic therapy (73).

It is important, however, that standardized large-scale trials to more thoroughly evaluate the role of probiotics in preventing NEC in the premature low birth weight population be done in an effort to solve unresolved issues mentioned above (104).

Chan et al. recently called “for more stringent regulations to hold the manufacturers of probiotics to safety standards similar to those with prescription medications so that well-designed observational studies to rigorously evaluate the safety of various strains and regimens can be conducted” (105). In USA, the FDA has published the Dietary Supplement Good Manufacturing Guidelines (http://www.fda.gov/Food/GuidanceRegulation/CGMP/ucm110858.htm), effective June 2008, which require that dietary supplements are manufactured consistently as to their identity, purity, strength, and composition. This is to ensure that consumers (i.e., NICUs in the present context) will have access to dietary supplements that meet quality standards that are free from contamination and are accurately labeled. The rule addresses the quality of manufacturing processes for dietary supplements and the accurate listing of supplement ingredients. However, this rule is currently not enforceable to the stringent standards of prescription medications, and is dependent on the manufacturer. The fact remains that there are already NICUs around the world that are/have been using probiotics to decrease rates of NEC, with published evidence-based guidelines (74), even as the multifaceted body of scientific literature surrounding their use grows.

## Directions for Further Research

As research surrounding the human microbiome continues to expand, we gather more information about its development and function that may ultimately assist us in a better understanding of lifelong disease processes. Because the genesis of the human microbiome is primarily influenced by factors external to the host, rather than intrinsic genetic factors, an understanding of these factors and how they can be manipulated could impact the balance between health and disease beginning at or even before birth. In time, we may be able to harness the power of this knowledge to guide newborn delivery decision making, and to target use of pre- and pro-biotic species to promote an as yet to be elucidated “healthy” gut milieu. These potential interventions could ultimately allow for prevention of and/or intervention against infectious and immune-mediated disease beginning as early as the neonatal period.

## Conflict of Interest Statement

The authors declare that the research was conducted in the absence of any commercial or financial relationships that could be construed as a potential conflict of interest.
