# Corrigendum: The Human Neonatal Gut Microbiome: A Brief Review

**DOI:** 10.3389/fped.2015.00060

**Published:** 2015-06-30

**Authors:** Emily C. Gritz, Vineet Bhandari

**Affiliations:** ^1^Division of Perinatal Medicine, Department of Pediatrics, Yale Child Health Research Center, Yale University School of Medicine, New Haven, CT, USA

**Keywords:** newborn, microbiome, gut, probiotics, necrotizing enterocolitis

There was an error in the nomenclature in original Figure 1. Members of the genus Ruminococcus belong to the phylum Firmicutes, not Proteobacteria. This has now been corrected in this revised figure.


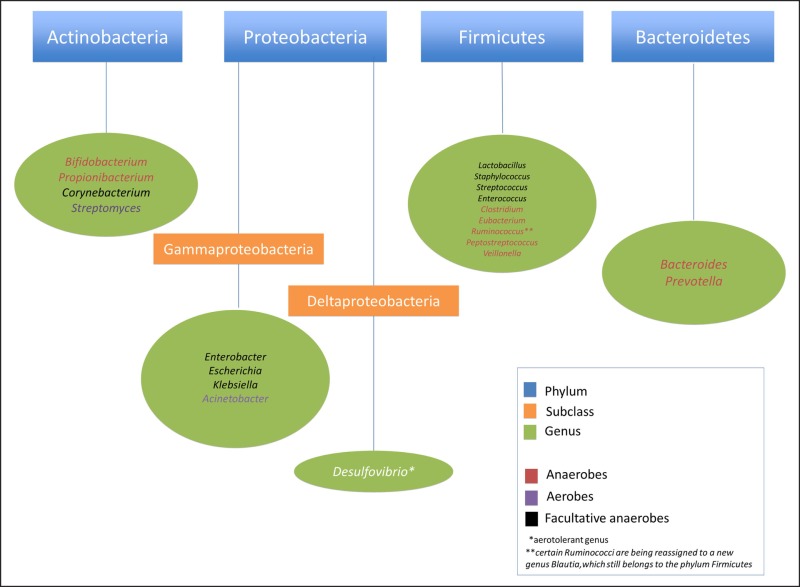


## Conflict of Interest Statement

The authors declare that the research was conducted in the absence of any commercial or financial relationships that could be construed as a potential conflict of interest.

